# Growth resumption from stationary phase reveals memory in *Escherichia coli* cultures

**DOI:** 10.1038/srep24055

**Published:** 2016-04-06

**Authors:** Arvi Jõers, Tanel Tenson

**Affiliations:** 1 Institute of Technology, University of Tartu, Tartu 50411, Estonia

## Abstract

Frequent changes in nutrient availability often result in repeated cycles of bacterial growth and dormancy. The timing of growth resumption can differ among isogenic cells and delayed growth resumption can lead to antibiotic tolerant persisters. Here we describe a correlation between the timing of entry into stationary phase and resuming growth in the next period of cell proliferation. *E. coli* cells can follow a last in first out rule: the last ones to shut down their metabolism in the beginning of stationary phase are the first to recover in response to nutrients. This memory effect can last for several days in stationary phase and is not influenced by environmental changes. We observe that the speed and heterogeneity of growth resumption depends on the carbon source. A good carbon source (glucose) can promote rapid growth resumption even at low concentrations, and is seen to act more like a signal than a growth substrate. Heterogeneous growth resumption can protect the population from adverse effect of stress, investigated here using heat-shock, because the stress-resilient dormant cells are always present.

Bacteria can encounter frequent changes in nutrient availability, often described as a feast-and-famine cycle. This results in constant cycling between dormancy and growth, making exit from dormancy an important and recurrent phase of the bacterial life cycle. Rapid growth resumption under favorable conditions should provide cells with an advantage over bacteria that recover slowly. Indeed, long-term evolutionary experiments have selected strains with a shorter lag phase that can outcompete ancestors in competition experiments^1^. However, wt *E. coli* strains display heterogeneity in growth resumption timing and many cells display a long lag phase^2^,^3^,^4^. This suggests that, despite favorable conditions, delaying growth resumption could be advantageous.

Dormant, nongrowing cells are more resilient to various stresses, including antibiotics and heat shock. Antibiotic-tolerant persister cells have been shown to be nongrowing even before exposure to antibiotics^5^ and slower growth resumption leads to more persisters^6^. A longer stationary phase results in more heterogeneous growth resumption^2^ and more persister cells^7^.

Delayed growth resumption has been suggested to be a bet-hedging type of behaviour^5^. Bet-hedging is an evolutionary strategy useful in the case of an uncertain future. A population of genetically similar organisms is split into two or more phenotypically different subpopulations, each optimal for different possible future environments. This ensures that at least some individuals are well positioned to thrive in an unknown future environment and population maximizes its geometric mean fitness over many generations. There is not enough evidence to claim that all known cases of heterogeneous growth resumption had evolved specifically as a bet-hedging behaviour^8^, however, experimental evidence in specific cases strongly support this view^9^,^10^.

During the heterogeneous growth resumption of the population the event of growth of the individual cell must be at least partially stochastic^11^ even if the distribution of growth resumption timing is set at the population level. Stationary phase cells are often considered to be similar to each other and no markers have been described that would predict the timing of regrowth of individual cells. Here we reassess this assumption. We show that under some conditions correlation exists between the timing of cell’s entrance into stationary phase and regrowth in the beginning of next growth phase. Cells that enter the dormant state late tend to be the ones that resume growth quickest when a new source of carbon becomes available. A correspondence between the status of each cell at the beginning of stationary phase and the timing of growth resumption is maintained over several days indicating the existence of long-term ‘memory’ in *E. coli* cultures.

## Results

### Heterogeneous growth resumption from stationary phase

**Figure 1:**
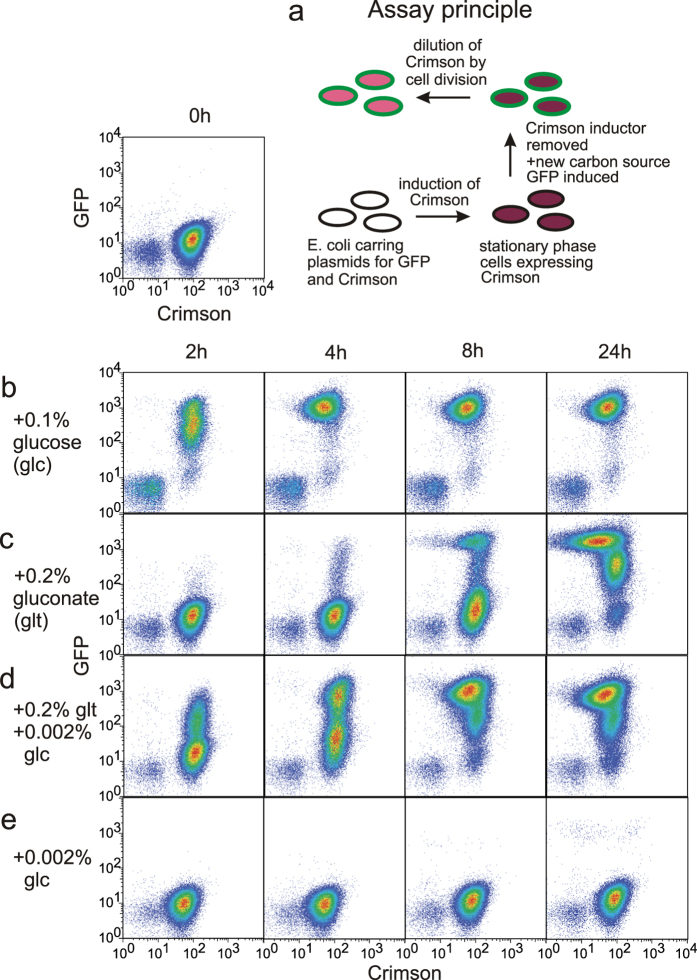
Heterogeneous growth resumption from stationary phase. (**a**) Assay principle. Cells with Crimson expression induced are grown until they reach stationary phase. Then Crimson inducer is removed and GFP inducer together with a new carbon source is added. Cells resuming growth at first become GFP-positive and later dilute Crimson by cell division. (**b–e**) Growth resumption in response to different carbon sourses. Cells were grown in MOPS 0.1% glycerol for three days and growth resumption was initiated by adding carbon sources indicated. Pseudocolours indicate the amount of cells.

When new growth resources become available to nutrient-limited stationary phase culture, cells resume cycles of growth and division. Previous reports^2^,^4^ have described heterogeneity in growth resumption and our original goal was to determine how the growth environment influences both the timing of growth resumption and phenotypic heterogeneity during it. We chose flow cytometry as a method for single cell analysis because it allows to analyze large amounts of cells and detect minor subpopulations. It also enables to grow cells in traditional liquid culture and sample it over time. For growth resumption analysis we developed a fluorescence-based two-color assay (Fig. 1a). *E. coli* cells, carrying two plasmids encoding for fluorescent proteins GFP and Crimson, are grown into stationary phase with Crimson expression induced. Then the Crimson inducer is removed and a new carbon source is added together with GFP inducer. Cells that resume growth, manifested by active protein synthesis, initially become GFP-positive and later dilute Crimson by cell division.

Adding glucose to stationary phase culture, grown in MOPS glycerol for 3 days, leads to rapid and homogeneous growth resumption (Fig. 1b). All cells become GFP-positive quickly and undergo one or two cell divisions before all glucose is consumed. However, when gluconate is added to stationary phase cells we observe considerable heterogeneity (Fig. 1c). Initially, only a small number of cells resume growth (start GFP synthesis) whereas the bulk of the culture becomes GFP-positive later. By that time the cells that resumed growth first have already divided several times, but some still remain in a dormant, GFP-negative state.

Growth resumption in response to gluconate can be significantly accelerated if a small amount of glucose is added together with gluconate (Fig. 1d). In this case glucose seems to “prime“ cells to exit dormancy and use gluconate as a carbon source. This small amount of glucose is not able to induce any significant protein synthesis by itself (Fig. 1e). In this experiment glucose acts in dose-dependent manner. Increasing glucose concentration speeds up the the growth resumption still dependent on gluconate (Supplementary Fig. S1).

Glucose is a preferred carbon source for *E. coli* and its presence inhibits the use of other carbon sources^12^, however glucose and gluconate can be co-metabolized^13^. The fact that glucose enhances, rather than postpones, growth resumption on gluconate suggests that catabolic repression does not control the timing of growth resumption. It also argues against the opinion that slowly recovering cells could be damaged and thus unable to rapidly resume growth^14^. If accumulated damage is the main determinant of the timing of growth resumption then it would have a similar effect under all conditions; in these experiments both the timing of growth resumption and phenotypic heterogeneity are highly sensitive to the recovery conditions.

Heterogeneous growth resumption is not determined by the choice of specific carbon sources, it can be induced by a variety of conditions (Supplementary Fig. S2).

### Prediction of growth resumption order of stationary phase cells

Heterogeneous growth resumption means that individual cells exit dormancy at different times. Growth resumption event could be a random, stochastic occurance that does not depend on the history of any particular cell. Alternatively, differences between individual cells in stationary phase could influence the timing of their growth resumption.

**Figure 2:**
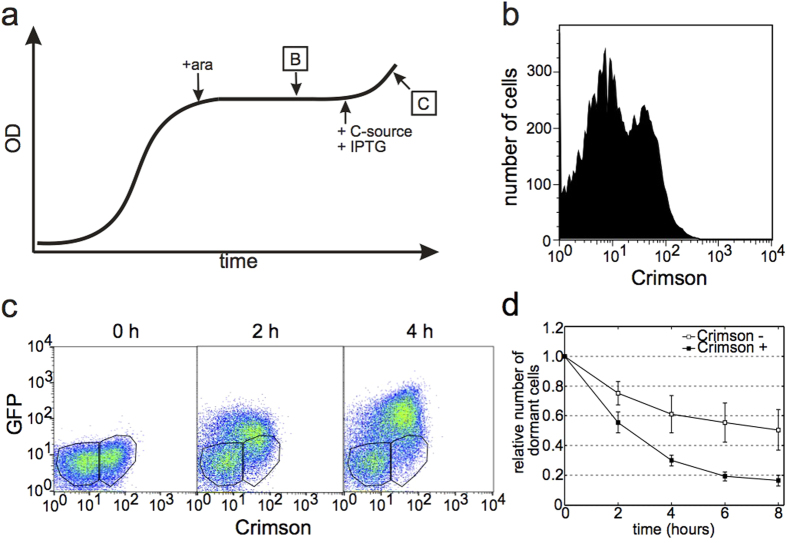
Growth resumption order depends on the entrance into stationary phase. **(a**) Experimental scheme to mark differences at the entrance into stationary phase. B and C refer to subsequent panels. (**b**) Crimson expression profile of stationary phase culture induced at the end of the growth phase. Two subpopulations are clearly distinguished from each other and the whole distribution is bimodal (Hartigan’s dip test for unimodality, D = 0.0061, p < 2.2 × 10^−16^). (**c**) Flow cytometry analysis of cells resuming growth. Crimson positive and Crimson negative subpopulations are gated separately for further analysis. (**d**) Growth resumption of cells from two different (Crimson-positive and Crimson-negative) subpopulation. Number of dormant (GFP-negative) cells is plotted at every timepoint. Values are an average from three independent experiments and error bars indicate s.e.m. Time-courses were analysed using linear regression t-test in Graphpad software package (GraphPad Software, USA) and found to be significantly different from each other (p = 0.0114).

In an attempt to discriminate between cells that display early and late growth resumption, we induced Crimson expression at different times during the first growth cycle. While most media tested led to a homogeneous stationary phase in terms of Crimson levels, the use of succinate as a carbon source resulted in heterogeneity. Adding the inducer during exponential growth uniformly induces Crimson expression in all cells, and if the inducer is added to stationary phase culture, no Crimson expression can be detected. However, if the inducer is added during the transition period from growth to stationary phase (Fig. 2a), heterogeneous expression of Crimson arises. In this case we observe two subpopulations: one with nondetectable Crimson levels and the other with clear Crimson expression (Fig. 2b). This bimodal expression suggests that during the transition from growth to stationary phase some cells shut down their metabolism earlier than others and are no longer able to respond to the Crimson inducer. Others are still active and capable of synthesizing Crimson when induced.

The bimodal expression pattern of Crimson stays unchanged over several days (Supplementary Fig. S3a) suggesting that Crimson is not degraded nor synthesized during the stationary phase. Addition of GFP inducer (IPTG) without a carbon source to stationary phase cells does not lead to any detectable GFP expression (Supplementary Fig. S3b) indicating the lack of metabolically active cells in these conditions. These results indicate that stationary phase in our experimental conditions is rather static and does not contain activly multiplying subpopulations that could take over the culture^15^. When growth resumption is initiated by addition of 0.2% gluconate, the two sub-populations behave differently. Cells, that maintained their activity for a longer period of time and became Crimson-positive, resume growth quicker than cells with no Crimson expression (Fig. 2c,d). Cells tend to follow a last in first out principle with regards to entering and exiting dormancy. The preference for Crimson-positive cells to exit dormancy first is not caused by Crimson expression as such – when mixing cells from fully induced and fully uninduced stationary phase cultures there is no preferential growth resumption of Crimson-positive cells (Supplementary Fig. S4).

Arabinose (ara), which is used to induce Crimson, is known to generate a positive feedback loop through the induction of its own transporter. This can lead to on-off type of regulation of ara-dependent promoters^16^. However, we still observe heterogeneous Crimson induction (Supplementary Fig. S5) using a strain where this positive feedback loop is eliminated^17^, which further supports our conclusion that metabolism shuts down unevenly at the end of the growth phase.

**Figure 3:**
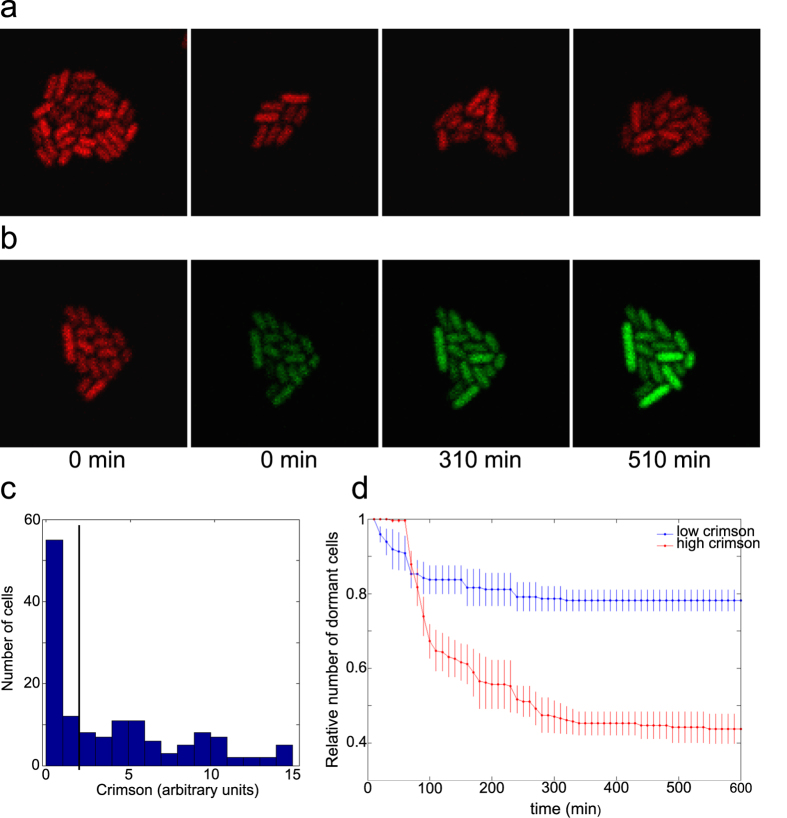
Microscopy analysis of growth resumption. **(a**) Examples of microcolonies exhibiting heterogeneous Crimson staining. (**b**) Growth resumption in microcolony after addition of gluconate and GFP inducer (IPTG). (**c**) Distribution of Crimson expression levels at 0 h timepoint. Black line separates low and high Crimson populations. **(d**) Growth resumption of cells with low and high Crimson content. Cells were grouped according to their Crimson expression levels at 0 h timepoint. Values are an average from three independent experiments and error bars indicate s.e.m. Time-courses were analysed using linear regression t-test in Graphpad software package and found to be significantly different from each other (p < 0.0001).

Essentially the same phenomenon is evident in microscopy studies. When cells are removed from the bulk culture at the end of growth phase and mounted on the agarose pad covered with cover glass they undergo a few divisions and form a microcolony. Crimson induction at that stage leads to heterogeneous expression (Fig. 3a). Adding a new carbon source together with inducer for GFP initiates growth resumption and GFP expression in heterogeneous manner (Fig. 3b).

We divided cells into low and high Crimson expressing sub-populations (Fig. 3c) and analysed their growth resumption dynamics separately. Similar to flow cytometry the increase of GFP signal over the background level was taken as an indicator for growth resumption. Two sub-populations showed different dynamics. High Crimson expressing cells recovered quickly and more than half of these became GFP-positive by the end of experiment (Fig. 3d). In contrast, low Crimson expressing cells resumed growth much slower and only about 20% of cells were able to express GFP by the end of experiment. This result was not sensitive to the exact value of the border between high and low Crimson expression; changing it did not change the pattern described in Fig. 3d. This confirms the results from flow cytometry experiments and further corroborates the correlation between cells’ condition during the entrance to and exit from stationary phase.

### Altered proportions of two subpopulations in Δ*relA* strain

(p)ppGpp has been implicated in persister formation and increased (p)ppGpp levels lead to cessation of growth^18^. In *E. coli* (p)ppGpp is synthesized by RelA and SpoT, RelA being responsible for an increase in (p)ppGpp levels during entrance into the stationary phase^19^. To test the role of (p)ppGpp in forming the last in first out phenomenon we analyzed a Δ*relA* strain by growing it in MOPS 0.1% succinate, inducing the Crimson expression at the end of the growth phase and adding 0.2% gluconate to stimulate growth resumption after three days in stationary phase.

**Figure 4:**
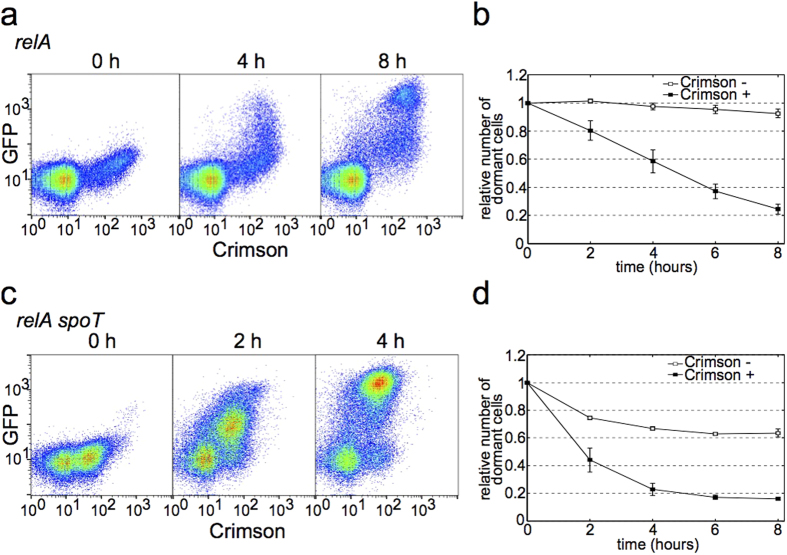
Growth resumption of strains with altered (p)ppGpp levels. **(a**) Stationary phase distribution of Crimson expression (0 h) and growth resumption in Δ*relA* strain. The distribution of Crimson expression in stationary phase is bimodal (Hartigan’s dip test for unimodality, D = 0.0104, p < 2.2 × 10^−16^). (**b**) Growth resumption timing correlates with Crimson expression levels in Δ*relA* strain. Time-courses were analysed using linear regression t-test in Graphpad software package and found to be significantly different from each other (p < 0.0001). (**c**) Stationary phase distribution of Crimson expression (0 h) and growth resumption in Δ*relA* Δ*spoT* strain. The distribution of Crimson expression in stationary phase is bimodal (Hartigan’s dip test for unimodality, D = 0.0175, p < 2.2 × 10^−16^). (**d**) Growth resumption timing correlates with Crimson expression levels in Δ*relA* Δ*spoT* strain. Time-courses were analysed using linear regression t-test in Graphpad software package and found to be significantly different from each other (p < 0.0001). Values are an average from three independent experiments and error bars indicate s.e.m.

Despite lacking the main (p)ppGpp synthesizing enzyme this strain still forms two subpopulations of Crimson negative and Crimson positive cells (Fig. 4a). However, the proportions of these two subpopulations are different from wt. In Δ*relA* strain the bulk of cells are Crimson negative and only a minority of cells become Crimson positive (less than 20%). In wt the ratio between the two subpopulations depends on the timing of Crimson induction, earlier addition of inducer results in higher number of Crimson positive cells. In Δ*relA* strain earlier addition of inducer leads to uniformly Crimson positive cells and when the heterogeneity appears the Crimson positive subpopulation is always much smaller than Crimson negative one.

Nevertheless, the Crimson positive subpopulation in Δ*relA* strain resumes growth quicker than Crimson negative (Fig. 4b). The latter is particulary slow in Δ*relA* strain and a very few cells resume growth from Crimson negative subpopulation during the experiment. This further underlines the well-described importance of (p)ppGpp role in stationary phase, but also confirms that in general wt and Δ*relA* strains are similar.

We also tested a Δ*relA* Δ*spoT* double mutant for subpopulation formation and growth resumption. These cells do not make any (p)ppGpp at all. Interestingly, this strain is very similar to wt - Crimson positive and negative populations are of the same size (Fig. 4c) and growth resumption dynamics is very similar to wt strain (Fig. 4d, compare to Fig. 2d). Our results suggest that dysregulation of (p)ppGpp has more dramatic phenotypic effect than the complete lack of it.

In the case of persisters, delayed growth resumption has been attributed to stochastic activation of toxins in toxin-antitoxin modules in some cells^18^,^20^. We analyzed the growth resumption heterogeneity of a mutant *E. coli* strain (Δ10TA), where 10 toxin-antitoxin modules have been eliminated^21^ (18), and found it indistiguishable from its isogenic wt (MG1655).

### Cellular ‘memory’ withstands stress and environmental change during stationary phase

Growth resumption order is established when cells enter stationary phase and this order is maintained during several days (Fig. 2). This could depend on external environment so that the stationary phase medium is needed for sustained last in first out dependency. Alternatively cells could be fixed to certain state and later changes in environment do not affect established growth resumption order. To distinguish between these two possibilities we replaced the growth medium in stationary phase cultures with water and tested the growth resumption.

**Figure 5:**
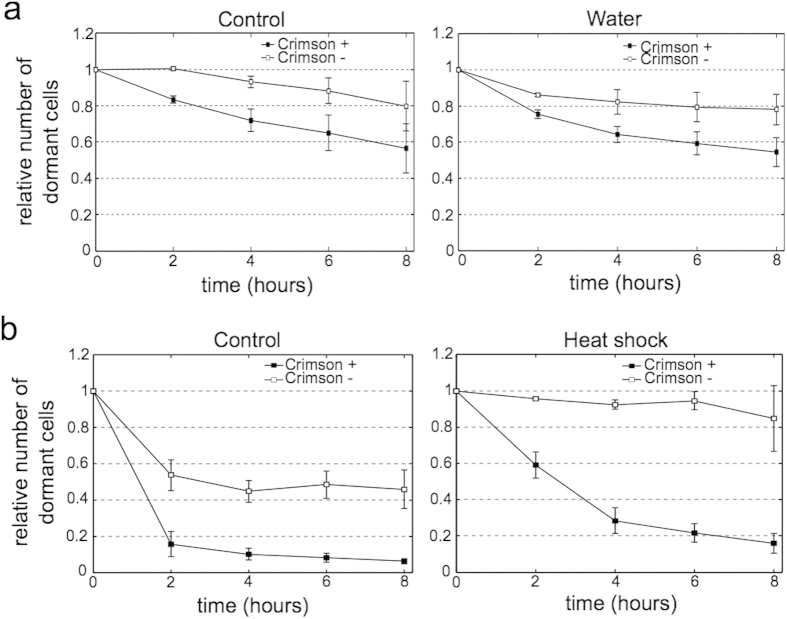
Change of enironment or heat-shock during the stationary phase does not change the order of growth resumption. **(a**) Growth resumption of cells in response to gluconate with or without replacing the stationary phase medium with water. Time-courses were analysed using linear regression t-test in Graphpad software package and found to be significantly different from each other (p = 0.0088 for control, p = 0.0402 for water). (**b**) Growth resumption in response to glucose with or without heat-shock. Time-courses were analysed using linear regression t-test in Graphpad software package and found to be significantly different from each other (p = 0.0012 for control, p < 0.0001 for heat-shock). Values are an average from three independent experiments and error bars indicate s.e.m.

Figure 5a shows no differences in growth resumption in cells held in water compared to control. Despite being exposed to different environment the last in first out phenomenon is still present and the overall dynamics is similar between these two cultures. This indicates that the inner state of the cells established in the beginning of stationary phase is stable and cannot be affected by replacing stationary phase medium with different enironment.

In addition to environmental change we tried to reset the cellular ‘memory’ by stressing the stationary phase cells with heat-shock. Although we were able to slow the growth resumption, the last in first out phenomenon still exists also after heat shock (Fig. 5b) and the difference between two subpopulations even increased.

We also tested if cells in different subpopulations respond in varying order to different carbon sources, however, we observed no such difference. Crimson positive cells were always the first to resume growth in response to various sugars, organic acids, and amino acids (Supplementary Table S1).

### Heterogeneous growth resumption as a possible insurance mechanism against lethal stress

Exiting dormancy and becoming metabolically active enables cells to multiply, however, this transition also makes cells more vulnerable to stress. Heterogeneous growth resumption could act as an insurance mechanism against potential hazards by allowing some cells to grow and multiply while others remain in dormancy to wait for a potential second chance. To test this hypothesis we compared the heat resistance of *E. coli* populations undergoing either homogeneous or heterogeneous growth resumption.

**Figure 6:**
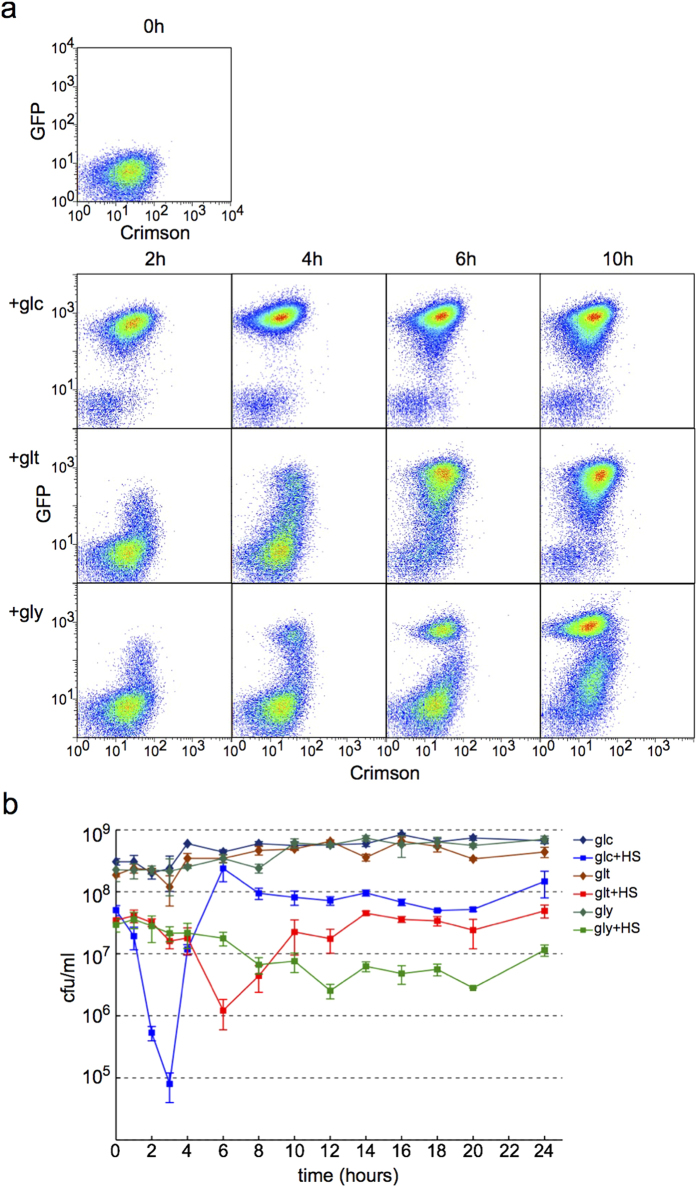
Heterogeneous growth resumption avoids periods of stress vulnerability. **(a**) Growth resumption is homogeneous in response to glucose and heterogeneous in response to gluconate and glycerol. Pseudocolours indicate cell density. (**b**) Heat sensitivity of cultures resuming growth. A small aliquote was removed from culture, heat-shocked (HS) for 5 minutes at 55 degrees and plated for cfu determination at indicated times. Samples without heat-shock were plated as controls at the same time. Values are an average from three independent experiments and error bars indicate s.e.m.

Cell growth was initiated by adding different carbon sources to two day old stationary phase culture in MOPS succinate . Glucose induced quick and homogeneous growth resumption (Fig. 6a, top row), gluconate, and more intensly glycerol led to a heterogeneous response (Fig. 6a, middle and bottom row). To determine heat-shock resistance a small aliquote was removed at each time point, heat shocked for 5 minutes at 55 °C, followed by measuring of CFUs. Regrowth on glucose caused the cells to became very sensitive to heat-shock 2–3 hours after growth initiation (Fig. 6b). This coincides with the period when cells are homogeneously resuming growth (Fig. 6a). The drop in viability is much smaller with gluconate and glycerol where growth resumption is heterogeneous and the population always contains dormant cells. These results corroborate the postulation that population heterogeneity acts as a protection mechanism against stress.

## Discussion

Some cells can significantly delay their growth resumption in a growth supporting environment, leading to the co-existance of growing and dormant cells^2^,^4^. Dormant cells in these populations can be identified as persisters: cells that are tolerant to antibiotics and can reinitiate growth when antibiotics are removed^5^. Here we analyzed the growth resumption dynamics directly and show that it is a continuous process (Fig. 1). Instead of splitting the culture into distinct ‘normal’ and ‘persister’ subpopulations the growth resumption can happen continuosly over extended time.

The process of growth resumption bears hallmarks of both stochastic and deterministic response. Cells of clonal origin in the same culture still resume growth at different times indicating that some stochastic process governs the exact timing of growth resumption. At the same time the dynamics of growth resumption depends on the carbon source, being quicker on glucose and slower on gluconate. Cells seem to sense the ‘quality’ of the carbon source and change the growth resumption accordingly. Such a mix of stochastic and responsive behavior has been described for sporulation in *Bacillus subtilis* - sporulation happens as a response to starvation, but never to all cells in population^22^,^23^. It has been shown theoretically that a mixture of stochastic and responsive behavior is optimal when environmental signals about future events are only partially reliable^24^.

Interestingly, a small amount of glucose can significantly accelerate growth resumption (Fig. 1d). Glucose seems to ‘kick-start’ cellular metabolism that will then consume gluconate for growth. Glucose could act here as a growth-promoting signal bearing information about the environment. Alternatively (or in parallel) cells could be energy-deprived and glucose might provide the necessary energy to switch to gluconate consumption. Small amount of glucose has been described to facilitate switch during diauxie^25^ and increasing the available energy facilitates growth during switch from glycolytic to gluconeogenetic carbon sources^26^.

Delayed growth resumption has been linked to stochastic activation of toxins in toxin-antitoxin modules in the case of persisters^18^,^20^. However, it is not known when this activation takes place or how many cells are involved. It is currently not possible to predict growth resumption timing for any one particular cell. We describe here a good correlation between the timing when metabolism is switched off at the end of the growth phase and the timing of resuming growth at the beginning of the next growth cycle. Cells tend to follow a last in first out principle where the last to halt growth are the first ones to recover. This allows us to mark these cells through reporter gene expression and generate markers for growth resumption timing.

Phenotypic heterogeneity is often considered to be a hallmark of bet-hedging. Indeed, more cells survive heat shock in heterogeneous culture where growing and dormant cells co-exist (Fig. 6). However, care must be taken to avoid overinterpretation^8^. Bet-hedging requires alternative phenotypes to confere different fitnesses across environments^27^ and phenotypes must interconvert between each other. Siegal and colleagues distinguish adaptive bet-hedging as a more strict term^27^ that should be used in cases where heterogeneity has been demonstrated to be a consequence of selection for distributed phenotypes. This is very difficult to be sure of and in the case of growth resumption described here we cannot claim it to be adaptive bet hedging.

Correlation between the state of the cells at the start and the end of stationary phase indicates the presence of a form of ‘memory’ in bacterial culture. Cells can ‘remember’ their status at the entrance into stationary phase which determines the timing of their growth resumption at the beginning of the next growth cycle. It is currently unclear what is the molecular basis of this ‘memory’, but the works of others suggest that cells in the same environment can exhibit metabolic variability^28^,^29^. Stochastic differences in the expression levels of a few genes can drive cells to different metabolic states that can co-exist in the same environment. This could lead to variability in metabolic shut-down at the start of the stationary phase and heterogeneous growth resumption while entering the next growth cycle. This ‘memory’ persists over several days in stationary phase and is not affected by changing conditions during that period. Additional stress during stationary phase, such as heat shock, does not weaken this memory, but rather makes it more pronounced; differences between subpopulations become more evident after heat shock (Fig. 5).

Another possible source of intrapopulation heterogeneity is asymmetric segregation of cell constituents during cell division. Cells receiving old poles during cell division have reduced growth rates^30^ and asymmetrically segregated protein aggregates are also associated with increased division times^31^. This leads to cells with different age - old cells have old poles and more protein aggregates. These differences, unavoidably occuring in the cell population, could form a basis for cellular ‘memory’ and be responsible for last in first out phenomenon. Cellular ‘memory’ has been described in the case of different *B. subtilis* developmental paths (growth/sporulation/lysis), although this was shown not to depend on cellular age but rather on phosphorylation of the sporulation master regulator^32^.

At the end of growth phase cells undergo big changes including transcriptional changes^33^ and ribosome degradation^34^,^35^. Any of these could be a sourse for observed heterogeneity, however the intrapopulation distribution of these changes is not known.

Early determination of subpopulations with different propensities for growth resumption suggests that *E. coli* is capable of anticipatory behaviour. As an *E. coli* population prepares for stationary phase, heterogeneity is preserved for later use. If conditions are favourable at the start of the next growth cycle, all cells rapidly recover and heterogeneity is masked. However, heterogeneity can become useful under less favourable conditions. Anticipatory behaviour has been described for *E. coli* in carbon source usage^36^ and here we report another example of cells that prepare for uncertain future environments.

## Methods

### Bacterial strains, plasmids and growth media

Unless noted otherwise, the experiments in this study were carried out using *E. coli* strain BW25113 (F-, Δ*(araD-araB)567*, Δ*lacZ4787*(::rrnB-3), *λ*-, *rph-1*, Δ*(rhaD-rhaB)568*, *hsdR514*). In addition strain MG1655 (F-, *λ*-, *rph-1*) was used. BW27786 is a derivative of BW25113^17^ where arabinose transporter is expressed constitutively in arabinose-independent manner. Δ10TA is a derivative of MG1655 where 10 toxin-antitoxin modules have been removed^21^.

Plasmid pET-GFP contains GFP under the control of a IPTG-inducible promoter and kanamycin resistance gene^37^. pBAD-Crimson (derivative of pBAD33) contains E2-Crimson gene^38^ under the control of arabinose-inducible promoter and chloramphennicol resistance gene. pC17-Crimson is derived from pBAD-Crimson by substituting *araC* coding sequence and *araBAD* promoter with a canonical *E. coli* promoter sequence ensuring constitutive expression (TGTTTAAAGATCCCCCCTCACTCCTGCCATAATTCTGAT**A**, −35 and −10 boxes underlined, the first transcribed nucleotide in bold). All the plasmids have medium copy-number. The presence of these plasmids does not hinder growth and plasmids are not lost during growth without antibiotics, indicating lack of noticable fitness cost (Fig. S6).

MOPS-based media substituted with different carbon sources^39^ were used in all experiments, supplemented with 25 μg/ml of kanamycin and chloramphenicol to ensure plasmid maintenance. In the case of Δ*relA* Δ*spoT* strain casamino acids (0.1 mg/ml) were added to growth medium. All experiments were initiated with frozen DMSO stock (8%).

### Growth resumption assay

Cells were grown at 37 °C on a shaker using conical flasks. Good aeration was required to achieve consistent results. For full Crimson induction (Fig. 1) 1 mM arabinose was added to the growth medium at the start of the experiment. An identical culture without arabinose was grown in parallel. To remove arabinose after cells had reached stationary phase, the cultures were centrifuged (5000 g) and supernatant from arabinose-containing culture was substituted with sterile-filtered arabinose-free supernatant.

To achieve heterogeneous Crimson induction 1mM arabinose was added at the end of growth phase at OD = 0.45–0.5 (0.5 is a plateau value; Fig. 2). For results described in Fig. 6 Crimson was expressed from pC17-Crimson constitutively.

Growth resumption was initiated after 2–3 days in culture by transfering 2 ml stationary phase culture into a test tube and adding a new carbon source together with 1 mM IPTG. Under these conditions, the amount of carbon source is growth-limiting and adding it is enough to stimulate growth. Samples for flow cytometry were taken at the times indicated, mixed 1:1 with 30% glycerol in PBS and stored at −70 °C pending analysis.

### Flow cytometry analysis

Flow cytometry analysis was carried out using LSRII (BD Biosciences) equipped with blue (488 nm) and red (638 nm) lasers. The detection windows for GFP and Crimson were 530 ± 15 nm and 660 ± 10 nm respectively. Flow cytometry samples stored at −70 °C were thawn at room temperature and 10 μl of cell suspension was diluted in 0.5 ml PBS to prepare for flow cytometer. Samples were analyzed at constant acquisition speed and for every sample events during 10 second time window were saved. This ensures the analysis of constant volume from each sample irrespective of the number of events registered resulting at least 30 000 events being analyzed per sample.

In each timeseries the 0 hour timepoint was used to gate GFP negative cells. Separate gates were drawn for Crimson negative and Crimson positive subpopulation (Fig. 2c). Gates were copied to the following timepoints in the same series and the number of cells in each timepoint in particular gate was recorded. For quantitative analysis the relative number of GFP negative cells was calculated for every timepoint (0 hour = 100%). The software packages FACSDiva and FlowJo were used to analyse flow cytometry data.

### Microscopy

Microscopy experiments were essentially done as in Young *et al*.^40^. Cells were seeded on the thin agarose pad (made with low melting agarose and MOPS medium without a carbon source), the pad was placed on the cover glass-bottom dish with bacteria between cover glass and agarose pad. Additional pieces of agarose were put inside glass-bottom dish to avoid drying and provide more humidity. Glass-bottom dish was put inside a bigger petri dish and incubated at 37 °C overnight. On the next day arabinose was added (1 mM) to induce Crimson expression. On the 3rd day glass-bottom petri dish was placed under the confocal imaging system (Zeiss LSM710) in environmental chamber preheated to 37 °C. For the quantitative analysis cells were grown in shake flask for 4 days and seeded on the agarose pad just before microscopy. Data were collected in the confocal mode during 10 hours in 10 minute intervals with autofocus on. At every timepoint signals in green (exitation 488 nm, emission 493–571 nm) and red(exitation 633 nm, emission 639–747 nm) channels were recorded for the whole visual field. The first 4 timepoints were recorded without inducing growth resumption or GFP induction and these were subsequently used to determine the baseline for GFP signal. Between the 4th and 5th timepoint gluconate (0.2%) and IPTG (1 mM) were added on the top of the agarose pad to initiate growth resuption.

Micriscopy pictures were analyzed using CellProfiler software^41^. Combined signal from green and red channels was used for automatic identication of cells and assignement them into timeseries (corrected manually if needed). Fluorescence intensity for each identified object was recorded. For further analysis custom written scripts in MATLAB (MathWorks) were used. GFP signal for every timeseries was normalised to the first timepoint (0 min) and the values for the first 4 timepoints were used to calculate the average and standard deviation for uninduced GFP signal. A threshold value for GFP positive cells was set to two standard deviations above the average and the time was recorded when this value was exceeded in the timeseries.

### Heat-shock sensitivity of cultures resuming growth

Growth resumption was initiated as described above and 100 μl samples were taken from the culture at indicated times. 10 μl was removed for plating and the remaining 90 μl was incubated for 5 minutes at 55 °C. Serial dilutions from both samples (before and after heat-shock) were spot plated onto LB agar plates and incubated at 37 °C overnight. Colonies were counted the next day.

## Additional Information

**How to cite this article**: Jõers, A. and Tenson, T. Growth resumption from stationary phase reveals memory in *Escherichia coli* cultures. *Sci. Rep*. **6**, 24055; doi: 10.1038/srep24055 (2016).

## Supplementary Material

Supplementary Information
