# Supplementary material for: Growth resumption from stationary phase reveals memory in Escherichia coli cultures
Source: Sci Rep. 2016 Apr 6;6:24055. doi: 10.1038/srep24055 (PMC4822139; doi:10.1038/srep24055)
Supplement: Supplementary Information [file srep24055-s1.pdf]

Supplementary Information:

Growth resumption from stationary phase reveals memory in *Escherichia coli* cultures.

Arvi Jõers*, arvi.joers@ut.ee, tel: +372 737 4846
Tanel Tenson, tanel.tenson@ut.ee, tel +372 737 4844

Institute of Technology, University of Tartu, Nooruse 1, Tartu 50411, Estonia.

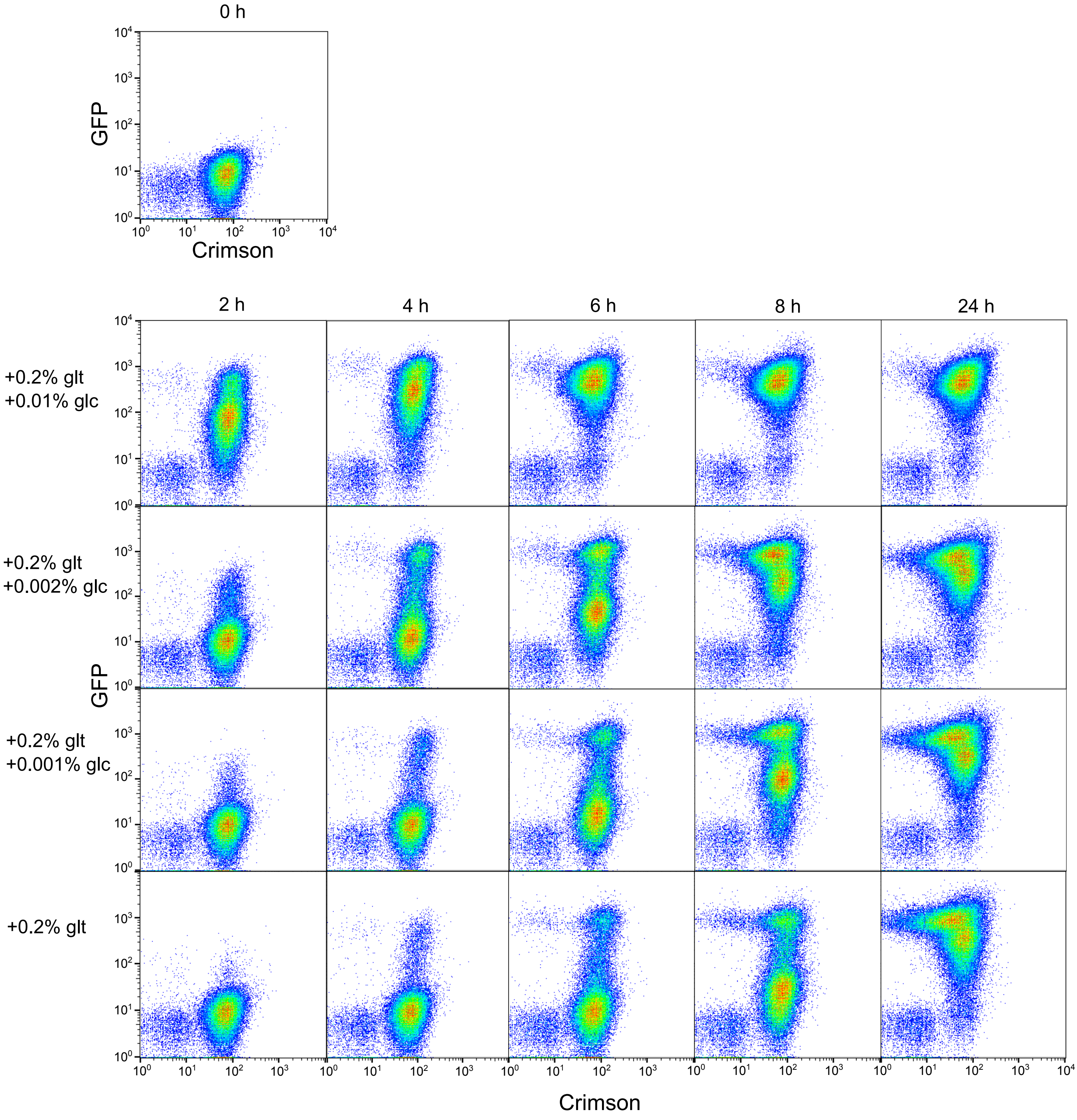

Figure S1. Glucose speeds up growth resumption based on gluconate in dose-dependent manner.

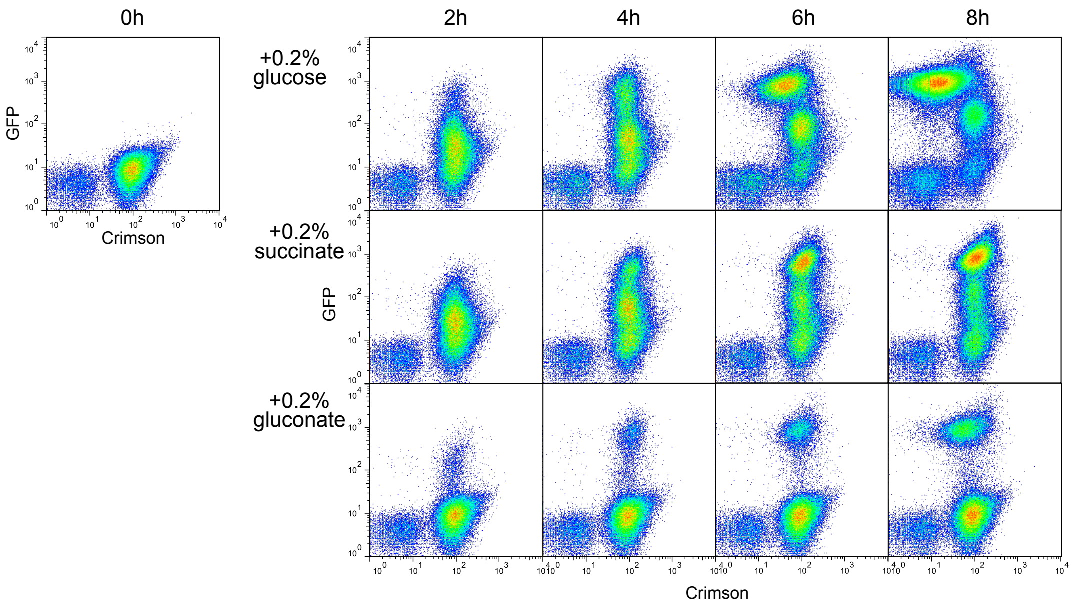

Figure S2. Growth resumption from stationary phase culture grown in MOPS succinate.

a

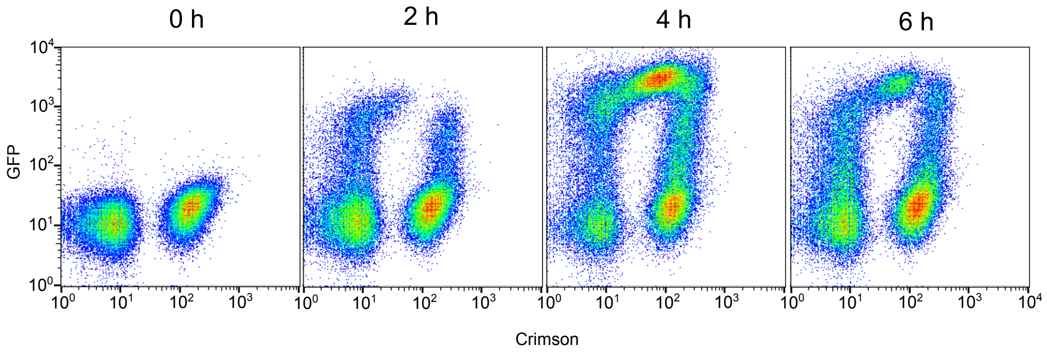

b

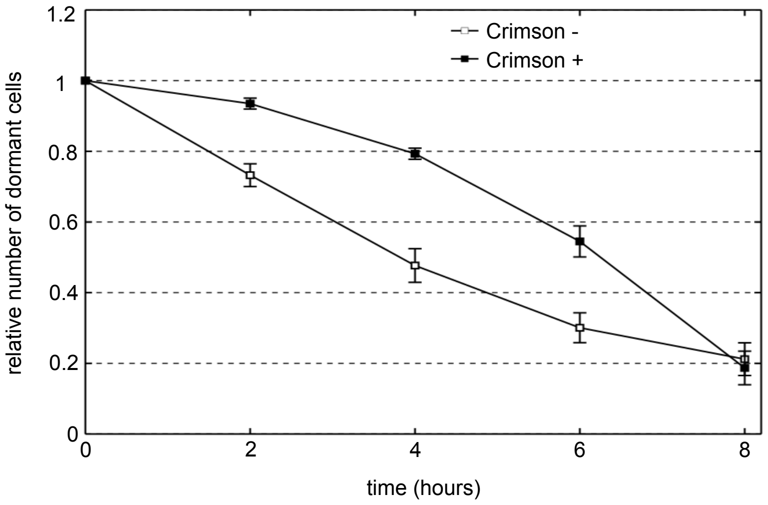

Figure S3. Crimson expression by itself does not promote growth resumption. Wt cells were grown in two separate cultures and Crimson expression was induced in one of them during exponential growth. In stationary phase two cultures were mixed and subjected to growth resumption assay. **a** - growth resumption of mixture of two cultures. **b** - growth resumption of cells from two different (Crimson-positive and Crimson-negative) subpopulation. Number of dormant (GFP-negative) cells are plotted at every timepoint. Values are an average from three independent experiments and error bars indicate s.e.m. Time-courses were analysed using linear regression t-test in Graphpad software package and found **not** to be significantly different from each other (p=0.4236).

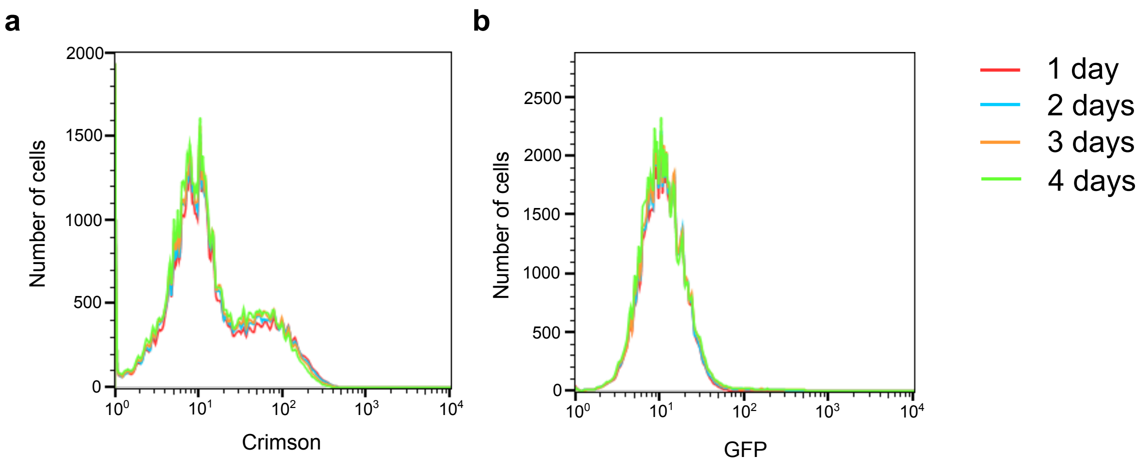

Figure S4. Crimson and GFP expression does not change during stationary phase. a - Crimson expression was induced at the end of growth phase. Samples were collected every day during the next 4 days of stationary phase and analysed on flow cytometer. No changes in Crimson expression pattern was seen during this period. **b** - Cells were grown into stationary phase and IPTG was added on day 1. Samples were collected during the next 4 days of stationary phase and analysed on flow cytometer. No GFP induction can be detected during the stationary phase.

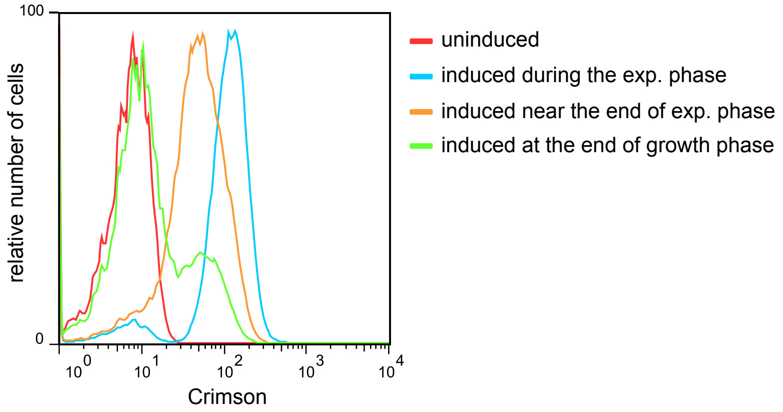

Figure S5. Bimodal Crimson induction in BW27786 strain. E. coli strain BW27786 was grown in MOPS 0.1% succinate. At different times 2 ml aliquote was removed from the main culture, Crimson expression induced and cultured separately. On the next day Crimson expression was analysed on flow cytometer. Despite uniform expression of arabinose transporter, bimodal Crimson expression can still be detected.

Supplementary table S1. List of carbon sources that result in heterogeneous growth resumption starting from a crimson-positive subpopulation.

Carbon source	Concentration
Succinate	0.1%
Acetate	0.1%
Galactose	0.1%
Gluconate	0.1%, 0.2%
Mix of 20 amino acids	100 µg/ml of each

A

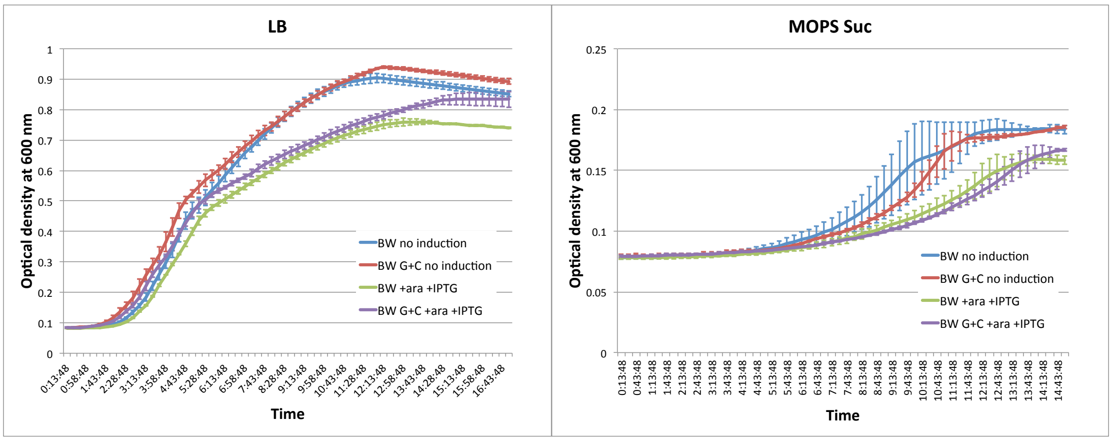

B

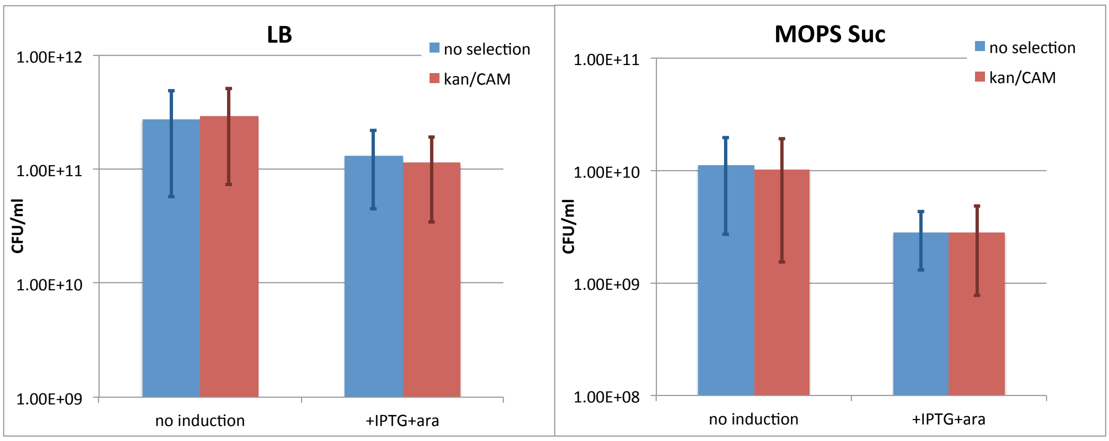

Figure S6. The presence of plasmids is not a significant burden for cells. A. Overnight cultures of BW25113 (BW) and BW25113 carrying plasmids pET-GFP and pBAD-Crimson (BW G+C) were grown in LB or LB containing kanamycin (kan) and chloramphenicol (CAM) respectively. At the start of an experiment 1:1000 dilution was made to LB and MOPS Suc without antibiotics. Cells were grown on 96-well plate and monitored with automatic plate reader. No significant differences exist between BW and BW G+C. Adding arabinose (ara) and IPTG to the cultures affects both strains in the same way regardless of the presence of the plasmids. The average of three independent experiments is shown, error bars indicate standard deviation. **B.** At the end of experiment described in panel A aliquots were taken from BW G+C cultures and plated on LB plates with and without antibiotics. The differences in colony forming units (CFU) between LB and LB kan/CAM plates would indicate plasmid loss, however no such differences exist. These results demonstrate that the presence of plasmids does not bear a noticeable fitness cost for cells and there is no selection for getting rid of these plasmids. The average of three independent experiments is shown, error bars indicate standard deviation.
